# Chemoenzymatic Synthesis and Antibody Binding of HIV-1 V1/V2 Glycopeptide-Bacteriophage Q*β* Conjugates as a Vaccine Candidate

**DOI:** 10.3390/ijms222212538

**Published:** 2021-11-21

**Authors:** Guanghui Zong, Christian Toonstra, Qiang Yang, Roushu Zhang, Lai-Xi Wang

**Affiliations:** Department of Chemistry and Biochemistry, University of Maryland, College Park, MD 20742, USA; gzong@umd.edu (G.Z.); toonstrac@gmail.com (C.T.); qyang1@umd.edu (Q.Y.); rzhang17@umd.edu (R.Z.)

**Keywords:** glycopeptide, glycoconjugate, neutralizing epitope, bacteriophage Q*β*, neutralizing antibody, HIV vaccine

## Abstract

The broadly neutralizing antibody PG9 recognizes a unique glycopeptide epitope in the V1V2 domain of HIV-1 gp120 envelope glycoprotein. The present study describes the design, synthesis, and antibody-binding analysis of HIV-1 V1V2 glycopeptide-Q*β* conjugates as a mimic of the proposed neutralizing epitope of PG9. The glycopeptides were synthesized using a highly efficient chemoenzymatic method. The alkyne-tagged glycopeptides were then conjugated to the recombinant bacteriophage (Q*β*), a virus-like nanoparticle, through a click reaction. Antibody-binding analysis indicated that the synthetic glycoconjugates showed significantly enhanced affinity for antibody PG9 compared with the monomeric glycopeptides. It was also shown that the affinity of the Q*β*-conjugates for antibody PG9 was dependent on the density of the glycopeptide antigen display. The glycopeptide-Q*β* conjugates synthesized represent a promising candidate of HIV-1 vaccine.

## 1. Introduction

A protective vaccine is the best hope to combat the global epidemic of HIV/AIDS. Despite tremendous efforts, an effective immunogen capable of eliciting long-lasting, broadly neutralizing antibodies (bNAbs) has yet to be realized [1,2,3,4,5,6,7,8,9,10]. HIV-1 has evolved a number of defense mechanisms, including frequent sequence variation and heavy glycosylation, to evade host immune surveillance [7,11,12]. For a long time, 2G12 that targets a cluster of high-mannose *N*-glycans in gp120 was the only carbohydrate-specific broadly neutralizing antibody available for HIV vaccine research [13,14,15,16,17]. However, in the last decade, a new class of glycan-reactive broadly neutralizing antibodies (bNAbs), represented by PG9, PG16, PGT121, PGT128, and PGT135 has been discovered, which appear to target conserved *N*-glycans and peptide as integral epitopes in the V1V2 and V3 regions [18,19,20,21,22,23,24]. These antibodies neutralize various HIV-1 primary strains with remarkable breadth and potency (up to 100-fold more potent than the prototype neutralizing antibodies such as 2G12 and 4E10). The discovery of this entirely new class of glycan-reactive bNAbs has raised new hopes and provided an enormous impetus to HIV vaccine research.

As the first step for an effective immunogen design, we recently applied a chemoenzymatic synthetic approach to characterize the glycan specificity and minimal glycopeptide neutralizing epitopes of the PG9, PGT128, PGT121, and 10-1074 bNAbs [25,26]. We synthesized a series of V1V2 glycopeptides with well-defined *N*-glycans at the conserved N160 and N156 (CAP45 strain) or N173 (ZM 109 strain) glycosylation sites. The study led to the identification of a cyclic glycopeptide carrying a Man_5_GlcNAc_2_ glycan and a sialylated complex type *N*-glycan at the N160 and N156/N173 sites as the minimal epitope for PG9 [25]. More recently, we synthesized a library of HIV-1 V3 glycopeptides derived from HIV-1 JR-FL strain and performed antibody binding studies, which identified a 33-mer mini-V3 glycopeptide carrying a high-mannose *N*-glycan at the N332 site as the epitope of bNAbs PGT128 and 10-1074, and the mini-V3 glycopeptide carrying a sialylated complex type *N*-glycan at the N301 site as a possible epitope for PGT121 [26]. Moreover, we designed and synthesized a self-adjuvant three-component immunogen that consists of the 33-mer V3 glycopeptide epitope and its trimeric form, a universal T-helper epitope P30, and a lipopeptide (Pam_3_CSK_4_) that is a ligand of Toll-like receptor 2 for stimulating immune response [27,28,29,30]. It was shown for the first time that the synthetic three-component glycopeptide immunogens can elicit glycan-dependent antibodies in a relatively short period of time. In addition, sera antibodies induced by the glycopeptide immunogen exhibited broad recognition of several HIV-1 gp120s across clades [27,28,29,30]. Independently, Haynes and coworkers have also reported that a 30-mer synthetic mini-V3 glycopeptide derived from the JR-FL strain, when formulated in the Toll-like receptor 4 agonist GLA-SE adjuvant, can induce V3-glycan targeted antibodies in rhesus macaques, although the monomeric V3 glycopeptide lacking T-helper epitopes are weakly immunogenic require high-dose and long repeated immunization to raise glycan-specific antibodies [31,32]. Despite these encouraging immunization results, the synthesis of the three-component glycopeptide immunogens takes multiple steps and is not easy to make. On the other hand, there are apparent advantages to use the virus-like particle (VLP), bacteriophage Q*β*, as a platform to design epitope-based vaccines [33,34]. The rigid and highly repetitive surface pattern permits highly ordered display of multiple copies of the glycopeptide epitopes, which could facilitate cross-linking of B-cell receptors and promote B cell activation. The highly repetitive pattern of epitope presentation on Q*β* may also enhance the avidity of the neutralizing epitopes for germ-line B cell receptors associated with the bNAbs, which is particularly important for HIV-1 vaccine design [35,36,37]. In comparison with gp120 and its modified derivatives that contain numerous non-neutralizing epitopes, the pure glycopeptide-based immunogen could focus immune responses to the glycopeptide neutralizing epitopes. Moreover, recent electron microscopy studies have suggested that the PG9, as other PG series bNAbs, is trimer-preferring, targeting an epitope at the apex of the gp120 trimer that spans across two monomers, as a second N160 glycan on a neighboring protomer was involved in PG9 recognition [38]. Thus, a vaccine design should consider the quaternary structure as an important factor for conjugate vaccine design. We reasoned that multivalent display of manifold, properly defined, glycopeptides on bacteriophage Q*β* could have a chance to generate mimic of the quaternary structure of the glycopeptide epitopes. Indeed, bacteriophage Q*β* has been successfully used as a carrier protein for carbohydrate-based cancer vaccine design [39,40,41,42]. Oligomannose-Q*β* conjugates have been synthesized before aiming to raise 2G12-like antibodies [43]. In this paper, we report the first chemoenzymatic synthesis of an array of V1V2 glycopeptide-Q*β* conjugates. Binding studies indicated that the conjugates carrying structurally well-defined N-glycans showed potent affinity for broadly neutralizing antibody PG9, which are comparable or superior to the affinity with native HIV-1 gp120. The results suggest that the synthetic conjugates may serve as a valuable HIV vaccine candidate for raising HIV-1 glycopeptide-specific antibodies.

## 2. Results and Discussion

### 2.1. Chemoenzymatic Synthesis of Alkyne-Tagged HIV-1 V1V2 Glycopeptides

**Scheme 1:**
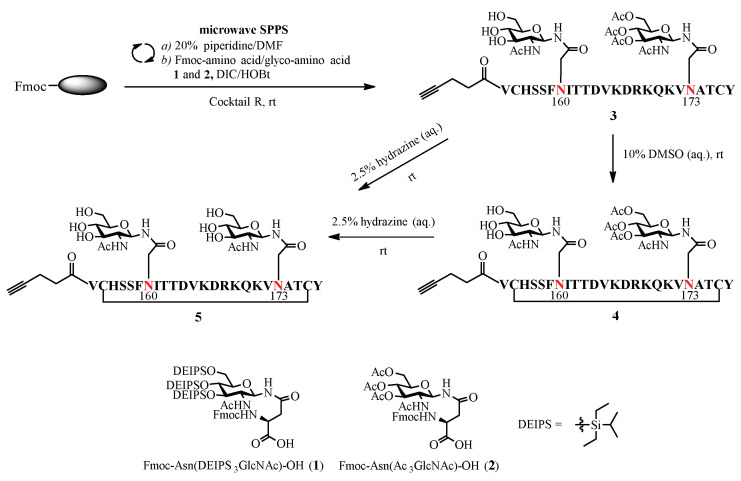
Synthesis of HIV-1 V1V2 GlcNAc peptides (**3**, **4**, **5**) through solid-phase peptide synthesis.

We have previously identified a cyclic glycopeptide epitope derived from the HIV-1 ZM109 strain carrying a Man_5_GlcNAc_2_ and a sialylated complex type N-glycan at the N160 and N173 site, respectively, as a minimal neutralizing epitope that demonstrate the specificity for antibody PG9, which demonstrate a μM-level affinity for the Fab domain of the antibody [25]. We chose this glycopeptide structure as the epitopic subunit and sought to introduce an alkyne moiety at the N-terminus of the peptide sequence for later stage Q*β* conjugation through a click reaction. The precursor GlcNAc-peptides were synthesized through microwave-assisted solid-phase peptide synthesis (MW-SPPS) using Fmoc chemistry on Rink Amide Resin. To install different glycans at the N160 and N173 sites, two orthogonally protected GlcNAc-Asn building blocks, i.e., acid-labile Fmoc-Asn(DEIPS_3_GlcNAc)-OH building block (**1**) and base-labile Fmoc-Asn-(Ac_3_GlcNAc)-OH building block (**2**) were installed at the predetermined *N*-glycosylation site N160 and N173, respectively, following our previously reported procedure [44] (Scheme 1). Global deprotection and simultaneous release of the peptide from the resin by treatment with cocktail R gave the precursor peptide (**3**), in which the silyl protection groups on the GlcNAc at position N160 was simultaneously removed, while the GlcNAc at N173 remained protected as the *O*-acetylated form. Cyclization via a disulfide bond formation between the two cysteine residues was achieved by treating **3** with 10% DMSO in water yielded cyclic GlcNAc-peptide **4** that are ready for introduction of two different *N*-glycans at the N160 and N173 sites. Removal of the *O*-acetyl groups from the remaining protected GlcNAc moiety with 2.5% aqueous hydrazine gave the peptide with two free GlcNAc acceptor sites (**5**). The di-GlcNAc peptide **5** was also obtained by direct treatment of peptide precursor **3** with 2.5% aqueous hydrazine to remove the *O*-acetyl groups from the GlcNAc moiety, which also led to simultaneous cyclization. The GlcNAc peptides were purified by reverse-phase HPLC (RP-HPLC). The purity and identity of the GlcNAc peptides were analyzed by RP-HPLC and ESI-MS (Supplementary Information, Figures S1–S4).

### 2.2. Chemoenzymatic Synthesis of the HIV-1 V1V2 Glycopeptides

**Scheme 2:**
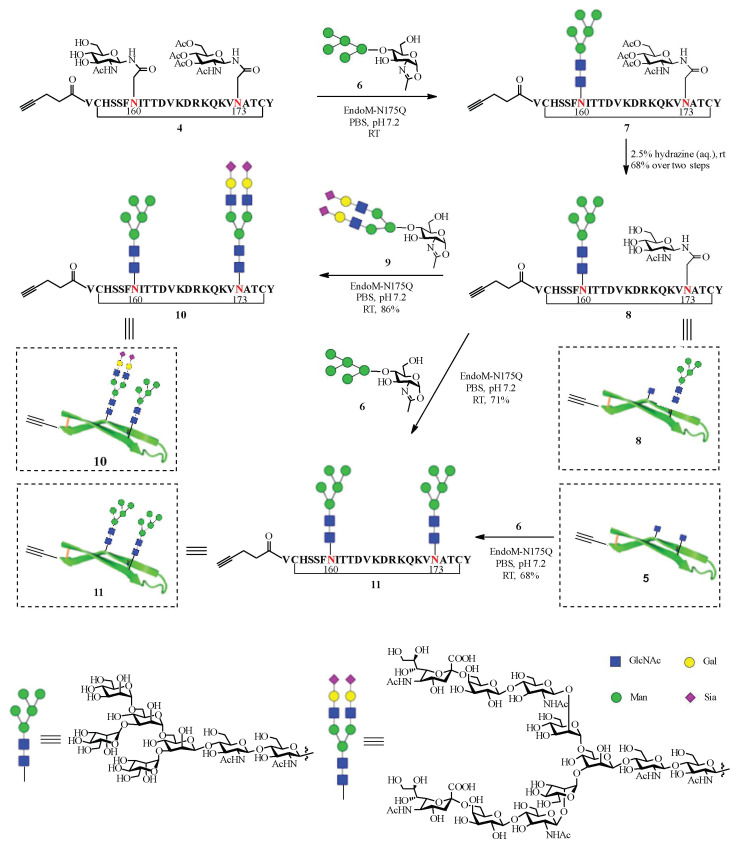
Chemoenzymatic synthesis of HIV-1 V1V2 glycopeptides (**8**, **10**, **11**).

**Figure 1:**
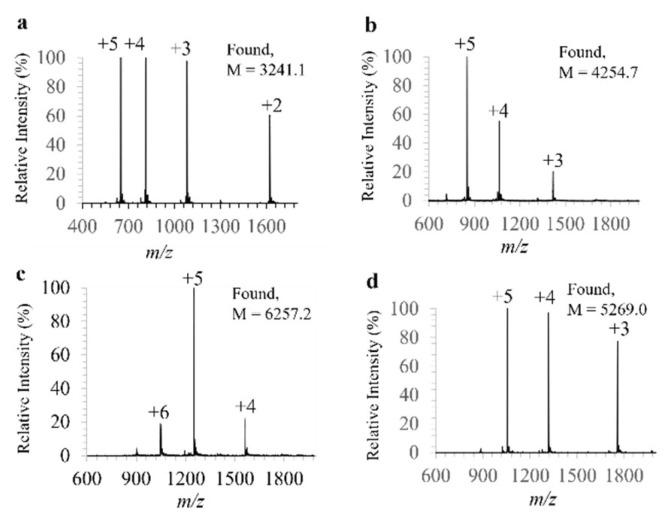
ESI-MS analysis of the synthetic glycopeptides. (**a**) glycopeptide **5**; (**b**) glycopeptide **8**; (**c**) glycopeptide **10**; (**d**) glycopeptide **11**.

With the selectively protected GlcNAc-peptides in hands, we first sought to introduce a Man_5_ glycan at the free GlcNAc moiety at the N160 site of GlcNAc-peptide **4**, using EndoM-N175Q mutant as the enzyme [45]. Thus, glycosylation of GlcNAc-peptide **4** with the Man_5_GlcNAc oxazoline **6** under the catalysis of EndoM-N175Q gave glycopeptide **7** carrying a Man_5_ glycan at the N160 site in excellent yield. The acetylated GlcNAc at the N173 position was then deprotected by treatment with 2.5% aqueous hydrazine, yielding glycopeptide **8** with the free GlcNAc moiety at the N173 site. Finally, a sialylated complex-type (SCT) *N*-glycan was installed on the free GlcNAc moiety by glycosylation with SCT-oxazoline **9** under the catalysis of EndoM-N175Q to give the doubly glycosylated peptide (**10**). To introduce two Man_5_GlcNAc glycans in the peptide, **8** was glycosylated with the Man_5_GlcNAc oxazoline **6** by EndoM-N175Q, giving the glycopeptide (**11**) carrying two Man_5_ glycans at both sites. Alternatively, glycosylation of GlcNAc-peptide (**5**) with excess of Man_5_GlcNAc oxazoline **6** under the catalysis of EndoM-N175Q- also gave glycopeptide **11** in good yield (Scheme 2). The transglycosylation products were purified by HPLC, and the purity and identity were confirmed by ESI-MS analysis (Figure 1; Supplementary Information, Figures S5–S8).

### 2.3. Synthesis and Characterization of Qβ-V1V2 Glycopeptide Conjugates

**Scheme 3:**
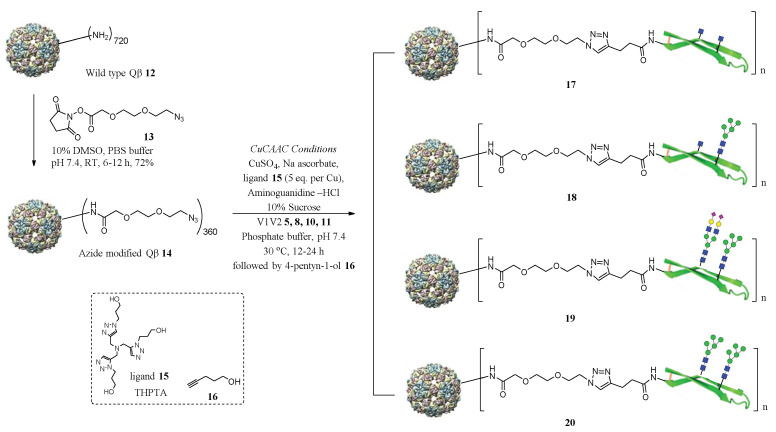
Synthesis of Q*β*-V1V2 glycopeptide conjugates **17**–**20**.

We initiated the Q*β*-V1V2 glycopeptide conjugates preparation by functionalization of wild type Q*β* with azido group. Firstly, we fully functionalized the lysine amines on Q*β* with a 5-carbon azido group by reaction with succinimidyl 5-azidovalerate, followed by ligation of the alkyne-tagged glycopeptides to the Q*β*-(5C-N_3_)_720_ via the copper-catalyzed azide-alkyne cycloaddition (CuAAC) reaction. However, the obtained Q*β*-V1V2 glycopeptide conjugates, especially the ones carrying large size glycopeptides (with two glycans) are liable to precipitation, if stored at 4 °C over a few days. The precipitation issue was not much applied to the conjugate with smaller glycopeptides (with one glycan or only GlcNAc). We speculated the reason for the poor stability of the conjugate might be, on one hand, the significant physical property changes of Q*β* caused by modification with a big molecule (M.W. of **10**: 6.3K, compared to 14K for Q*β* subunit); on the other hand, the overall lipophilicity increased dramatically by transforming the free amines on wild type Q*β* to triazoles after functionalization and ligation. To solve the problems, we replaced the lipophilic 5C-azide linker to a more hydrophilic polyethylene glycol (PEG) linker to increase the solubility in water. Meanwhile, we decreased the azide loading to keep around half of the lysine amines free to better maintain the physical property of Q*β*. As shown in Scheme 3, functionalization of wild type Q*β* (**12**) with N_3_-PEG_3_-NHS (**13**) to get Q*β*-PEG_3_-N_3_ with an average azide loading of 360 (estimated by MALDI-TOF analysis). Incubation of Q*β*-PEG_3_-N_3_ with alkyne-tagged V1V2 glycopeptides (**5**, **8**, **10**, **11**) via CuAAC yielded the corresponding Q*β*-V1V2 glycopeptide conjugates **17**–**20** (Scheme 3). 10% of sucrose was added in the CuAAC incubation to avoid potential protein aggregation [46]. The glycopeptide loading level was controlled by the amount of alkyne-tagged glycopeptides used (ratio of glycopeptides/subunit) and the incubation time. To get a high loading of the glycopeptides onto Q*β* (1–2 glycopeptides/subunit), 3–5 eq of alkyne-tagged glycopeptides were used, and the incubation was last for up to 24 h. The unreacted azido groups on Q*β* capsids were capped using a large excess of 4-pentyn-1-ol (**16**) by a second CuAAC reaction. The Q*β*-V1V2 glycopeptide conjugates were purified by dialysis (MWCO 300K membrane) against 150 mM PBS buffer.

**Figure 2:**
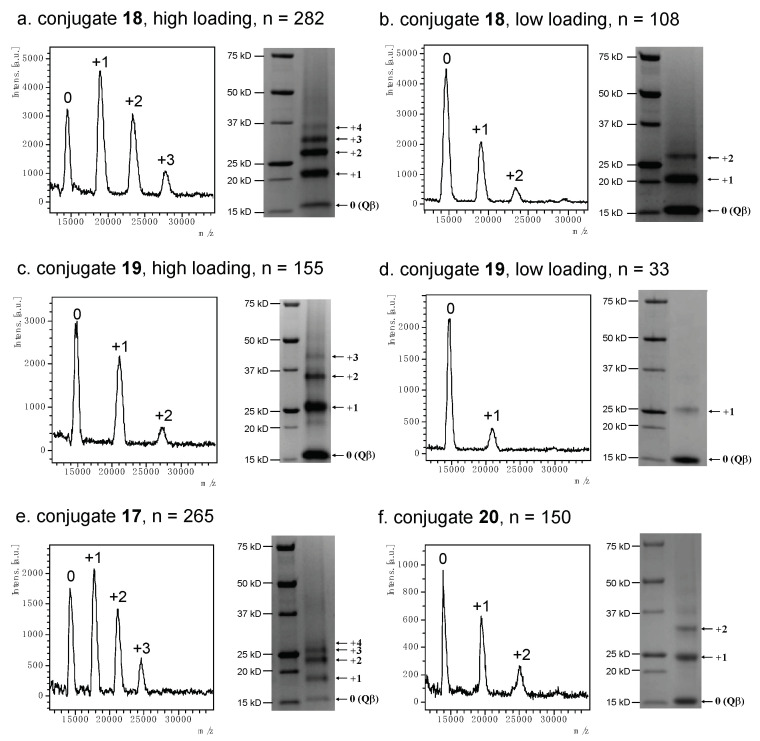
MALDI-TOF MS and reducing SDS-PAGE analysis of Q*β*-V1V2 glycopeptide conjugates **17**–**20** (**a**), conjugate **18** with high loading; (**b**), conjugate **18** with low loading; (**c**), conjugate **19** with high loading; (**d**), conjugate **19** with low loading; (**e**), conjugate **17** with high loading; (**f**), conjugate **20** with high loading.

To determine the glycopeptides loading, we performed both MALDI-TOF MS analysis and SDS-PAGE quantification. Overall, MALDI-TOF MS and SDS-PAGE analysis gave a similar estimate of the glycopeptide loading ratios (Figure 2). SDS-PAGE analysis exhibited the average numbers of (glyco)peptides attached onto Q*β* were 282, 265, 155 and 150 for conjugates **17**, **18**, **19** and **20**, respectively (Figure 2e, Figure 2a, Figure 2c and Figure 2f, respectively).

### 2.4. Binding of the Synthetic Conjugates with Antibody PG9

To assess the binding of the synthetic glycopeptide-Q*β* conjugates to antibody PG9, we used conventional ELISA and compared the binding with HIV-1 gp120 envelope glycoproteins from clade M, strain M.CON-S (gp120_M_._CON-S_) and AE.A244.

**Figure 3:**
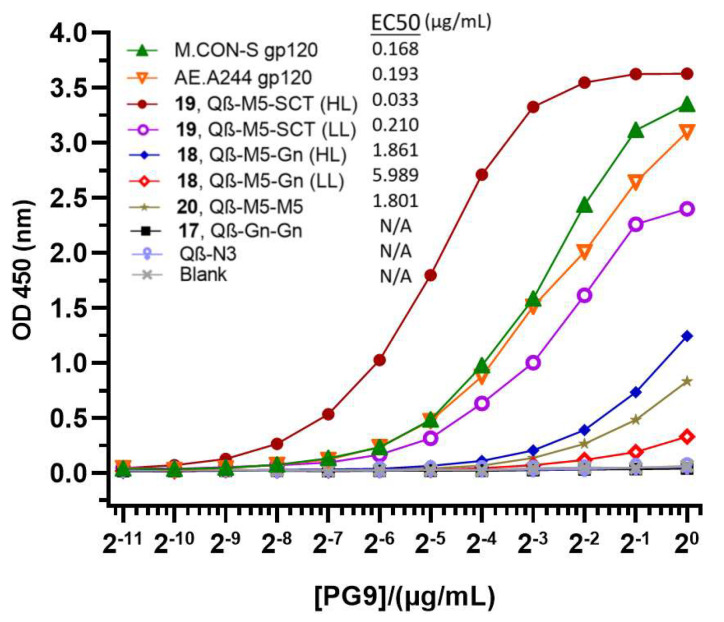
Conventional ELISA binding of V1V2 antigens to bNAb PG9. Q*β*-V1V2 conjugates or gp120 were coated on the microtiter plates. PG9 was titrated with two-fold serial dilutions starting from 1 μg/mL. LL = low-loading (below 1 peptide/subunit); HL = high-loading (1–2 peptides/subunit); Blank is PBS buffer. Two independent ELISA assays were performed and the results were consistent. The figure shows a representative set of the data.

Antigenicity of the conjugates was found to be highly dependent upon glycosylation, as conjugates bearing two GlcNAc alone (**17**, Q*β*-Gn-Gn) yielded no recognition by PG9 (Figure 3), whereas the conjugates bearing one or two glycans (**18**, **19**, **20**) substantially increased the binding affinity. As a negative control, nonconjugated Q*β* did not show affinity for PG9. The results suggest that the nonglycosylated V1V2 peptides is insufficient for significant binding affinity by PG9, even in a multivalent context. The finding is in an agreement with the results of PG9 binding to monovalent peptides containing GlcNAc alone, reported previously by our group and others [25,44,47]. The previous reports indicated no binding affinity by PG9 for nonglycosylated putative V1V2 peptides.

The addition of a single Man_5_GlcNAc_2_ glycan (conjugate **18**) significantly increased the binding affinity by PG9 (EC_50_ = ~12.4 nM, Figure 3, Q*β*-M5-Gn, HL), compared to its nonglycosylated V1V2 peptide-Q*β* counterpart (conjugate **17**), suggesting that the glycan at N160 site is crucial for the PG9 recognition. The conjugate bearing both glycans (**19**, Figure 3, Q*β*-M5-SCT, HL), i.e., Man_5_GlcNAc_2_ at N160 site and SCT-GlcNAc_2_ at N173 site, exhibited a subnanomolar level binding affinity (EC_50_ = ~0.20 nM). Although the loading level for conjugate **19** (loading *n* = 155) is lower than that for conjugate **18** (loading *n* = 265), a 62-fold enhancement in binding affinity for PG9 was observed. Of note, the binding affinity of conjugate **19** for PG9 was significantly higher than that of gp120_M_._CON-S_ (EC_50_ = ~1.12 nM) and gp120_AE_._A244_ (~1.29 nM) monomer, suggesting that the multivalent display provides enhanced binding, when compared to monomeric gp120. The conjugate **20** bearing Man_5_GlcNAc_2_ glycan at both N160 and N173 positions with glycopeptide loading of 150 showed an EC_50_ of ~12.0 nM.

To compare the effects of glycopeptide antigen loading on the affinity, we also synthesized a panel of Q*β* conjugates with relatively low loading by reducing the amounts of alkyne-tagged glycopeptides (1–2 eq were used) and shortening the incubation time (3–12 h). Thus, conjugate **18** bearing 108 copies of glycopeptide **8** and conjugate **19** bearing 33 copies of glycopeptide **10** (Figure 2b,d, respectively) was prepared. The low-loading glycopeptide-Q*β* conjugate **18** exhibited ~3.2-fold reduced binding affinity (EC_50_ ≈ 39.9 nM, Figure 3, Q*β*-M5-Gn, LL) for PG9 compared to the high-loading conjugate (Figure 3, Q*β*-M5-Gn, HL). Similarly, low-loading glycopeptide-Q*β* conjugate **19** exhibited ~7.0-fold reduced binding affinity (EC_50_ ≈ 1.4 nM, Figure 3, Q*β*-M5-SCT, LL) for PG9 compared to the high-loading conjugate (Figure 3, Q*β*-M5-SCT, HL). These data suggest that an appropriate dense presentation of the glycopeptide epitope on the Q*β* nanoparticles are important to achieve the high-affinity binding to the broadly neutralizing antibody PG9. Future studies will be directed to immunization to evaluate the immunogenicity of the glycoconjugates.

## 3. Materials and Methods

### 3.1. General Procedures

Analytical reverse-phase HPLC was performed on a Waters Alliance^®^ e2695 HPLC system equipped with a dual absorbance 2489 UV/Vis detector. Separations were performed using a C18 column (YMC-Triart C18, 4.6 × 250 mm, 5 μm) at a flow rate of 1 mL/min using a linear gradient of 5–40% MeCN containing 0.1% FA over 30 min. ESI-MS spectra were obtained using a Waters SQ Detector 2 single quadrupole mass spectrometer. MALDI-TOF analysis was performed using a Bruker UltrafleXtreme (UTX) mass spectrometer with TOF/TOF detection. Preparative RP-HPLC was performed on a Waters 600 HPLC system equipped with a dual absorbance UV detector using a C18 column (Waters-Symmetry Prep C18, 19 × 300 mm, 7 μm) at a flow-rate of 10 mL/min, or a C18 column (Waters XBridge, Prep Shield, 10 × 250 mm, 5 μm) at a flow-rate of 4 mL/min. The column was eluted using a linear gradient of 10–30% MeCN containing 0.1% FA over 30 min.

### 3.2. Peptide Synthesis

Peptide synthesis was performed under microwave synthesis conditions using a CEM Liberty Blue microwave peptide synthesizer. Synthesis was based on Fmoc chemistry using Rink Amide resin (0.66 mmol/g) on a 0.1 mmol scale. Couplings were performed using 5 equiv of Fmoc-protected amino acids (or 3 equiv of glycosylamino acids), 5 equiv of DIC and 5 equiv of HOBt in DMF. Double couplings were performed at 45 °C for 20 min (2×) for Fmoc-Cys(Trt)-OH, Fmoc-His(Trt)-OH and Fmoc-Arg(Pbf)-OH. The selectively protected glycosylamino acid building block Fmoc-Asn(Ac_3_GlcNAc)-OH (**1**) and Fmoc-Asn(DEIPS_3_GlcNAc)-OH (**2**) was introduced at predetermined sites and the coupling was performed at 45 °C for 40 min. All other amino acids were coupled at 90 °C for 2 min. Fmoc deblocking was performed in 20% piperidine in DMF containing 0.1 M HOBt. The N-terminus was capped with an alkyne tag by treatment with 4-pentynoic acid following the same method as introduction of glycosylamino acid. After synthesis, the resin was washed with DMF (3×) and DCM (3×), dried. Resin cleavage and global peptide deprotection were achieved using freshly prepared cocktail R (TFA/Thioanisole/Ethanedithiol/Anisole [90/5/3/2]) and shaking for 2 h. The peptide was separated from the resin by filtration and the peptide was precipitated onto cold (−20 °C) diethyl ether. The precipitated crude GlcNAc-peptide **3** was not further purified and was used directly for the synthesis of GlcNAc-peptide **4** and **5**.

#### 3.2.1. Synthesis of GlcNAc-Peptide **4** Bearing a GlcNAc at Asn160 and a Ac_3_GlcNAc at Asn173

A solution of the crude GlcNAc-peptide **3** (0.02 mmol) in water (45 mL, final concentration: 1–2 mg/mL) was added DMSO (5 mL). The mixture was shaken at rt for 24 h to cyclize the peptide. The crude peptides were purified by RP-HPLC, yielding the target glycopeptide **4** as a white powder (37.7 mg, 56% overall yield from SPPS). Analytical RP-HPLC, *t*_R_ = 16.57 min. ESI-MS: calcd., M = 3367.7; found, 842.7 [M+4H]^4+^, 1123.51 [M+3H]^3+^, 1684.47 [M+2H]^2+^. Deconvolution mass, 3367.5.

#### 3.2.2. Synthesis of GlcNAc-Peptide **5** Bearing a GlcNAc at Asn160 and a GlcNAc at Asn173

A solution of the crude GlcNAc-peptide **3** (0.02 mmol) in 2.5% aq. hydrazine (final concentration: 1–2 mg/mL) was shaken at rt for 3 h to cyclize the peptide and to remove the acetyl protecting group simultaneously. The crude peptides were purified by RP-HPLC, yielding the target GlcNAc-peptide **5 [48]** as a white powder (39.5 mg, 61% overall yield from SPPS). Analytical RP-HPLC, *t*_R_ = 14.57 min. ESI-MS: calcd., M = 3241.6; found, 649.04 [M+5H]^5+^, 811.22 [M+4H]^4+^, 1081.68 [M+3H]^3+^, 1621.72 [M+2H]^2+^ Deconvolution mass, 3241.4.

### 3.3. EndoM Mutant-Catalyzed Transglycosylation

#### 3.3.1. Synthesis of Glycopeptide **8** Bearing a Man_5_GlcNAc_2_ Glycan at Asn160 and a GlcNAc at Asn173

EndoM-N175Q mediated transglycosylation of Man_5_GlcNAc oxazoline **6** to GlcNAc-peptide was performed following a similar procedure as we have previously reported [44]. A solution of glycopeptide **4** (6.0 mg, 1.8 μmol) and Man_5_GlcNAc oxazoline **6** (14.5 mg, 14.3 μmol, 8 eq) in a phosphate buffer (100 mM, pH 7.2, 0.3 mL) was incubated with EndoM mutant N175Q (final concentration, 0.1 mg/mL) at 30 °C. The reaction was monitored by RP-HPLC-MS. After 2 h, the reaction was quenched with 0.1% aq. TFA. The mixture was centrifuged and filtered through a 0.45 μm syringe filter. The filtrate was purified by RP-HPLC to give glycopeptide **7**. The lyophilized glycopeptide **7** was dissolved in 2% aq. hydrazine (1 mL) and the solution was shaken for 1 h for deacetylation. The mixture was centrifuged and filtered through a 0.45 μm syringe filter. The filtrate was purified by RP-HPLC, yielding the target glycopeptide **8** as a white powder (5.5 mg, 68%). Analytical RP-HPLC, *t*_R_ = 14.18 min. ESI-MS: calcd., M = 4255.5; found, 851.94 [M+5H]^5+^, 1064.63 [M+4H]^4+^, 1419.32 [M+3H]^3+^. Deconvolution mass, 4254.7.

#### 3.3.2. Synthesis of Glycopeptide **10** Bearing a Man_5_GlcNAc_2_ Glycan at Asn160 and a Sialylated Complex-Type Glycan (SCT) at Asn173

EndoM-N175Q mediated transglycosylation of complex-type N-glycan oxazoline to GlcNAc-peptide was performed following a similar procedure as we previously reported [44,49,50]. A solution of glycopeptide **8** (3.0 mg, 0.70 μmol) and SCT-oxazoline **9** [44] (11.3 mg, 5.6 μmol, 4 eq) in a phosphate buffer (100 mM, pH 7.2, 0.15 mL) was incubated with EndoM mutant N175Q (final concentration, 0.1 mg/mL) at 30 °C. The reaction was monitored by RP-HPLC-MS. After 1 h, the reaction was quenched with 0.1% aq. TFA. The mixture was centrifuged and filtered through a 0.45 μm syringe filter. The filtrate was purified by RP-HPLC, yielding the target glycopeptide **10** as a white powder (4.4 mg, 86%). Analytical RP-HPLC, *t*_R_ = 15.10 min. ESI-MS: calcd., M = 6258.3; found, 1043.91 [M+6H]^6+^, 1252.44 [M+5H]^5+^, 1465.48 [M+4H]^4+^. Deconvolution mass, 6257.2.

#### 3.3.3. Synthesis of Glycopeptide **11** Bearing a Man_5_GlcNAc_2_ Glycan at Asn160 and a Man_5_GlcNAc_2_ Glycan at Asn173

A solution of glycopeptide **5** (5.0 mg, 1.5 μmol) and Man_5_GlcNAc oxazoline **6** (12.5 mg, 12.3 μmol, 8 eq) in a phosphate buffer (100 mM, pH 7.2, 0.25 mL) was incubated with EndoM mutant N175Q (final concentration, 0.1 mg/mL) at 30 °C. The reaction was monitored by RP-HPLC-MS. After 2 h, the reaction was quenched with 0.1% aq. TFA. The mixture was centrifuged and filtered through a 0.45 μm syringe filter. The filtrate was purified by RP-HPLC, yielding the target glycopeptide **11** as a white powder (5.5 mg, 68%). Alternatively, glycopeptide **11** (3.5 mg, 71%) was also obtained by transferring Man_5_GlcNAc oxazoline **6** (3.8 mg, 3.8 μmol, 4 eq) to glycopeptide **5** (4.0 mg, 0.94 μmol) following the above condition. Analytical RP-HPLC, *t*_R_ = 13.67 min. ESI-MS: calcd., M = 5269.4; found, 1054.97 [M+5H]^5+^, 1318.25 [M+4H]^4+^, 1757.36 [M+3H]^3+^. Deconvolution mass, 5269.0.

### 3.4. Expression and Functionalization of Bacteriophage Qβ Particles

#### 3.4.1. Expression of Wild Type Bacteriophage Q*β*

The virus-like nanoparticle Q*β* was expressed as previously described [51]. The BL21(DE3) E. coli strain was transformed with the plasmid encoding the Q*β* subunit. The clones were picked and verified. The expression of the Q*β* was induced with IPTG and performed at 25 °C. The bacteria cells were harvested and resuspended into PBS buffer (pH 7.4) and then lysed by ultrasonication (Branson digital sonifier). The Q*β* VLP was purified from the cell lysate through a Sepharose CL-4B (GE Healthcare Life Sciences) size exclusion column. Fractions were confirmed and characterized by MALDI-TOF MS and electrophoresis analysis. The protein was quantified by NanoDrop at 280 nm with an E% of 26.3.

#### 3.4.2. Functionalization of WT Q*β*

WT Q*β* (**12**, 45 mg) in PBS buffer (150 mM, pH 7.4, final protein concentration: 4 mg/mL) was incubated with NHS-activated compound **13** (12 equivalents) at 37 °C for 3 h. The progress of the functionalization of WT Q*β* was monitored by MALDI-TOF analysis. The reaction mixture was transferred into a dialysis bag (MWCO 10 KDa), and the solution was dialyzed against deionized water (2L × 3). The product could be concentrated by centrifugal filtration using Millipore 10 KDa MWCO filters if needed. The product was quantified by NanoDrop at 280 nm with an E% of 26.3. Average loading of surface azido groups was estimated by MALDI-TOF analysis.

### 3.5. Conjugation of V1V2 Glycopeptides to Qβ through CuAAC

#### 3.5.1. General Reaction Conditions for CuAAC 

Synthesis of glycopeptide-Q*β* conjugates (**17**–**20**) was achieved using the CuAAC click reaction, with conditions similar to those previously reported [52]. All solvents were degassed before use. In general, the following reagents were combined in the following order, 2 mg **14** (3 mg/mL stock in water), 1 M phosphate buffer (pH 7.4, final 80 mM), alkyne-tagged glycopeptides (**5, 8, 10, 11**) [2–3 eq./subunit], 50% aq. sucrose (final 10%), pre-mixed Cu-THPTA solution (CuSO_4_, 500 mM in water [4 eq.] + THPTA ligand **15**, 1 M in water, [20 eq.]), aminoguanidine (1 M in water, 100 eq. compared to **14**), and sodium ascorbate (1 M in water, 100 eq. compared to **14**). After the addition of sodium ascorbate, the reaction tube was flushed with argon and sealed. The mixture was shaken at 37 °C overnight. The low-loading glycopeptide-Q*β* conjugates were synthesized by a similar method, however, 0.5–1 eq./subunit of **8** or **10** was added to achieve low-loading. The remaining unreacted azido groups on Q*β* virion were capped in a second CuAAC reaction using a large excess (100 eq. compared to **14**) of 4-pentyn-1-ol (**16**) for a further 3 h. Purification of the glycopeptide-Q*β* conjugates was performed by dialysis against PBS buffer (50 mM, pH 7.4) using a dialysis bag (MWCO 300 KDa, BIOTECH CE TRIAL^®^, Repligen, Waltham, MA, USA). The product was quantified by NanoDrop at 280 nm with an E% of 26.3. Average loading of surface glycopeptides was estimated by reducing SDS-PAGE analysis.

#### 3.5.2. SDS-PAGE Sample Preparation and Analysis

Approximately 3 μg of the dialyzed glycopeptide-Q*β* conjugate or the conjugation reaction mixture diluted in water was mixed with 5 μL of 2X SDS loading dye containing *β*-mercaptoethanol to make a final volume of 10 μL. The samples were denatured at 95 °C for 5 min and cooled to 4 °C before loading. Precast polyacrylamide stain-free gels 4–15% were used for electrophoresis (Mini-PROTEAN™ TGX Stain-Free™ Protein Gels, Bio-Rad, Hercules, CA, USA, Cat. # 4568086). 6 μL of protein ladder (Precision Plus Protein Standards, Bio-Rad, Hercules, CA, USA, Cat. # 1610363) was loaded for reference. The gels were run in 1X Tris/Glycine/SDS buffer at 220 V for 35 min and stained with Coomassie Brilliant Blue R-250 Staining (Amresco, Solon, OH, USA), then destain the gel with destaining solution (40% methanol and 10% glacial acetic acid). Gel imaging was done using Gel Doc™ EZ Imager (Bio-Rad, Hercules, CA, USA).

#### 3.5.3. MALDI-TOF MS Sample Preparation and Analysis

The V1V2-derivitized Q*β* antigens (2 μg) were mixed with dithiothreitol (DTT) (final concentration 0.1 M). The mixture was incubated at 37 °C for 1 h. The resulting samples (1 μL) were mixed 1:1 with sinapic acid (SA) solution (50:50 water/acetonitrile with 0.1% TFA) onto the MALDI plate and allow to air dry. A second SA (1 μL) matrix was added on the top of the dried sample and allow to air dry.

### 3.6. ELISA Analysis of the Binding between PG9 and Glycopeptide-Qβ Conjugate

The high-binding Ultra Cruz 96-well plates (Santa Cruz Biotech, Dallas, TX, USA) were firstly coated with 100 ng of the Q*β* antigen or gp120 in 100 μL of PBS buffer at 4 °C overnight. Afterwards, the plates were washed with PBS/0.05% Tween-20 (PBS-T) three times and then saturated with 2% (*w/v*%) sodium casein under incubation at 37 °C for 1 h. The plates were again washed with PBS-T for three times. Next, the PG9 bNAb with various concentrations (1 μg/mL to 0.5 ng/mL with a serial of two-fold dilutions) was applied to the assay plate. The binding of the bNAb was proceeded at room temperature for 1 h and then washed by the PBS-T solution four times. Subsequently, 100 μL of the 1:10,000 diluted HRP-labeled goat anti-human IgG antibody (KPL) was applied to the well plate. The binding occurred at room temperature for 30 min, followed by extensive washes with PBS-T solution five times. Finally, 100 μL the TMB 2-component microwell peroxidase substrates (SeraCare, Milford, MA, USA) was added to each well. The color was developed in dark at room temperature for 30 min, and then quenched with 100 μL of the 1 M H_3_PO_4_ solution. Final results were read by subtracting the optical density (OD) at 450 nm by the OD at 550 nm.

## 4. Conclusions

An efficient chemoenzymatic synthesis of HIV-1 glycopeptide-Q*β* conjugates is described. The ratio of glycopeptide epitope loading on the Q*β* bacteriophage can be modulated by adjusting the ratios of tag introduction into Q*β* followed by a click reaction. The conjugates demonstrate much enhanced affinity for neutralizing antibody PG9 over monomeric envelope glycoprotein gp120. This study suggests that an appropriate multivalent display of the structurally well-defined glycopeptide epitope might catch the tertiary conformational epitopes of PG9. Thus, the synthetic HIV-1 glycopeptide-Q*β* conjugates represent a valuable HIV-1 vaccine candidate, the immunogenicity of which should be evaluated in animal models.

## Supplementary Materials

The following are available online at https://www.mdpi.com/article/10.3390/ijms222212538/s1.
