# Supplementary material for: Chemoenzymatic Synthesis and Antibody Binding of HIV-1 V1/V2 Glycopeptide-Bacteriophage Qβ Conjugates as a Vaccine Candidate
Source: Int J Mol Sci. 2021 Nov 21;22(22):12538. doi: 10.3390/ijms222212538 (PMC8617853; doi:10.3390/ijms222212538)

Supporting Information

Chemoenzymatic Synthesis and Antibody Binding of HIV-1 V1/V2 Glycopeptide-Bacteriophage Q*β* Conjugates as a Vaccine Candidate

Guanghui Zong [1,†], Christian Toonstra [1,†], Qiang Yang [1], Roushu Zhang [1] and Lai-Xi Wang [1,*]

[1] Department of Chemistry and Biochemistry, University of Maryland, College Park, Maryland 20742, United States

† Equal contributions as co-first authors

* Correspondence: wang518@umd.edu; Tel.: +1-301-405-7527 (L.X.W.)

Table of Contents

1. RP-HPLC and LC/MS Profiles S2-12

RP-HPLC and ESI-MS Profiles of (Glyco)peptides

HPLC conditions:

Analytical reverse-phase HPLC was performed on a Waters Alliance® e2695 HPLC system equipped with a dual absorbance 2489 UV/Vis detector. Separations were performed using a C18 column (YMC-Triart C18, 4.6 × 250 mm, 5 μm) at a flow rate of 1 mL/min using a linear gradient of 5-40% MeCN containing 0.1% FA over 30 min. ESI-MS spectra were obtained using a Waters SQ Detector 2 single quadrupole mass spectrometer.

Figure S1. HPLC profile of cyclic GlcNAc-peptide **4**

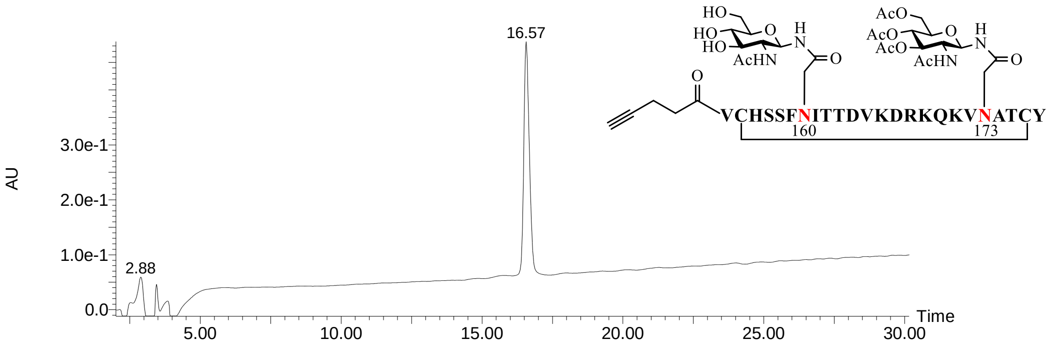

A. (UV Abs. 214 nm)

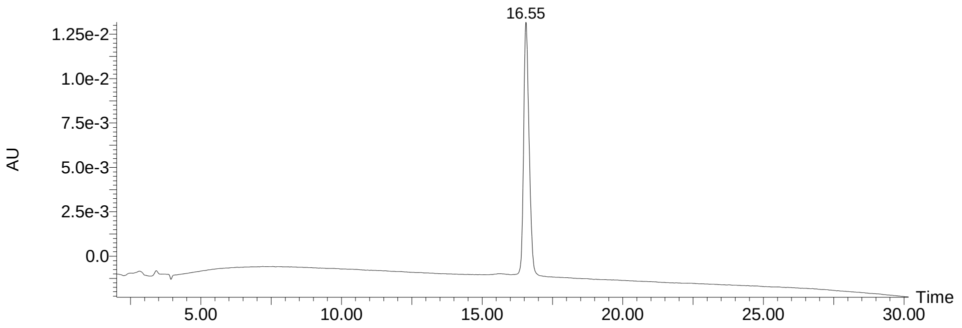

B. (UV Abs. 280 nm)

Figure S2. ESI-MS profile of cyclic GlcNAc-peptide **4**

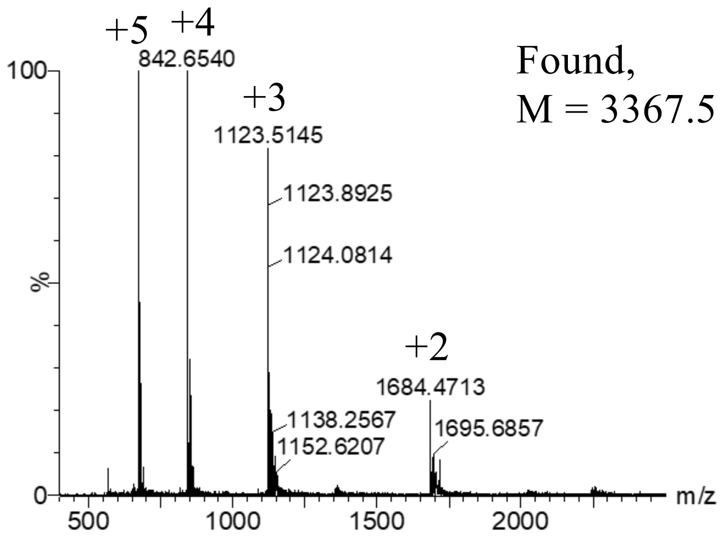

Figure S3. HPLC profile of cyclic GlcNAc-peptide **5**

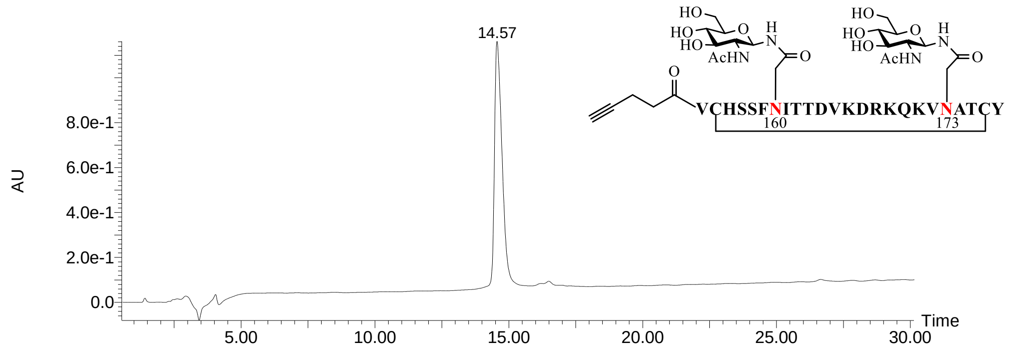

A. (UV Abs. 214 nm)

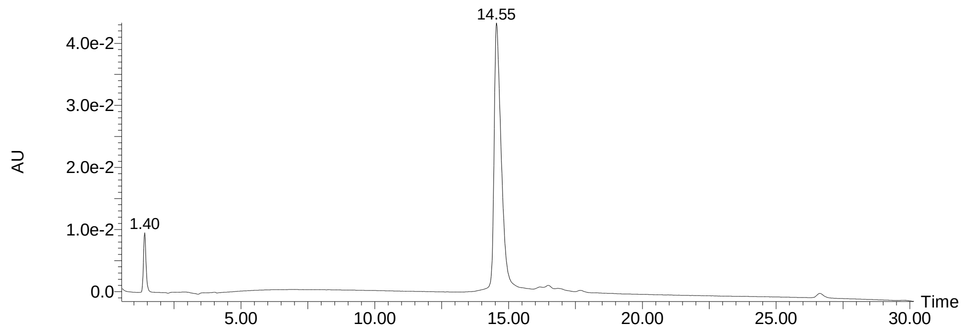

B. (UV Abs. 280 nm)

Figure S4. ESI-MS profile of cyclic GlcNAc-peptide **5**

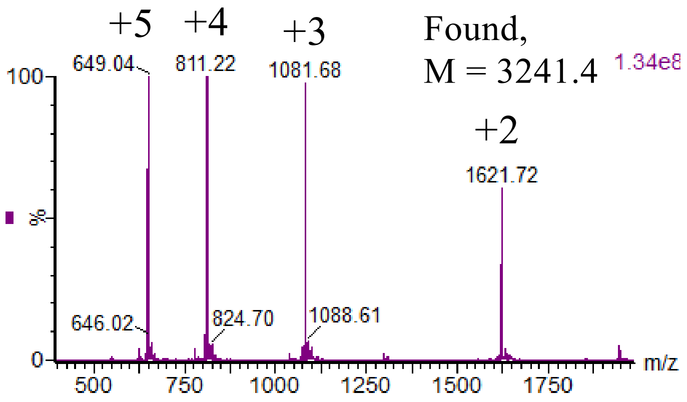

Figure S5. HPLC profile of cyclic GlcNAc-peptide **8**

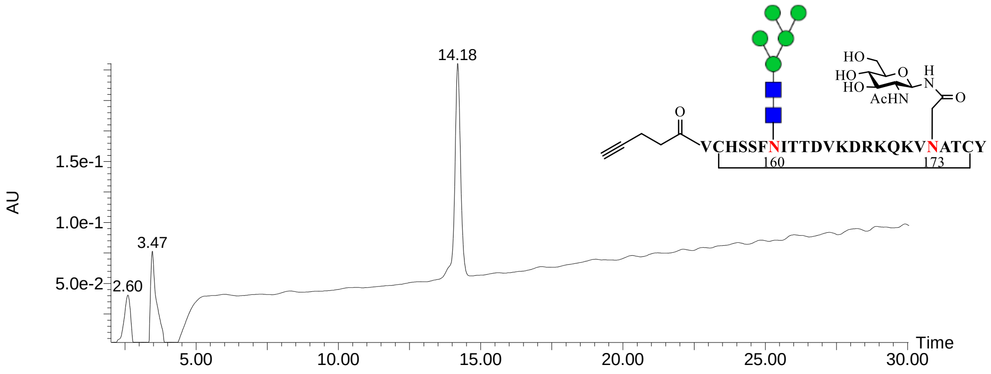

A. (UV Abs. 214 nm)

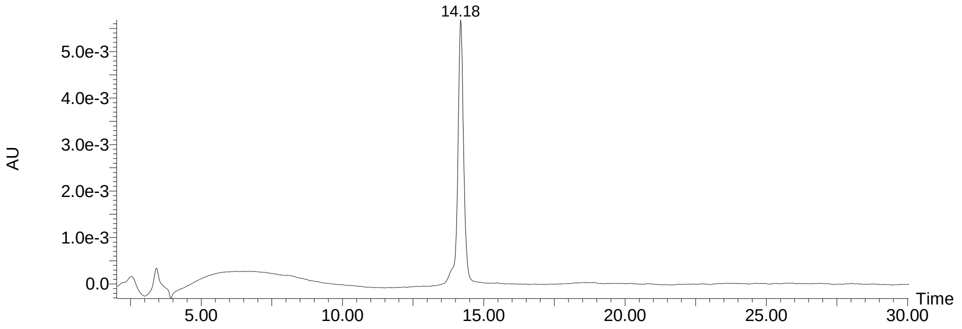

B. (UV Abs. 280 nm)

Figure S6. ESI-MS profile of cyclic glycopeptide **8**

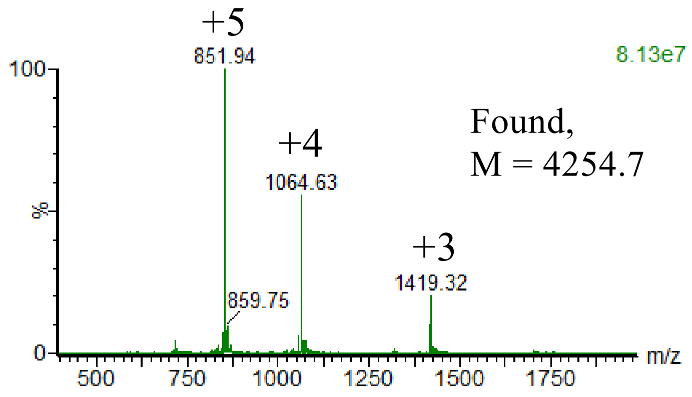

Figure S7. HPLC profile of cyclic glycopeptide **10**

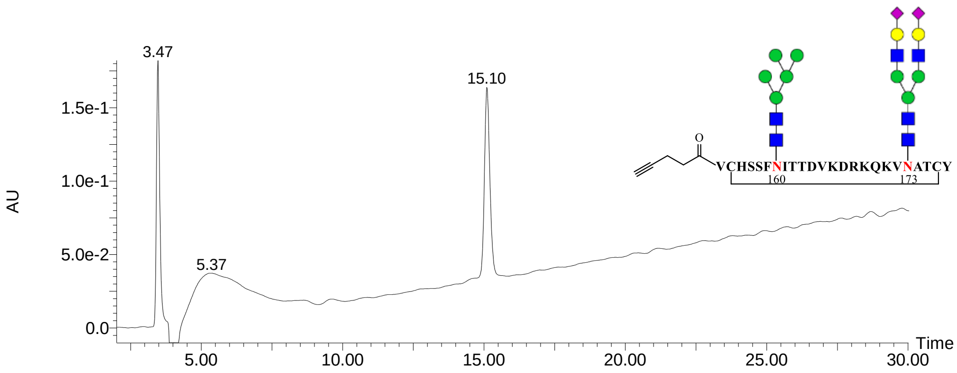

A. (UV Abs. 214 nm)

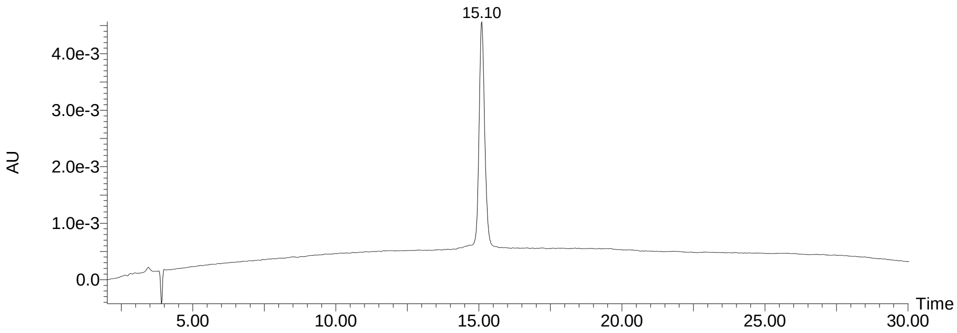

B. (UV Abs. 280 nm)

Figure S8. ESI-MS profile of cyclic glycopeptide **10**

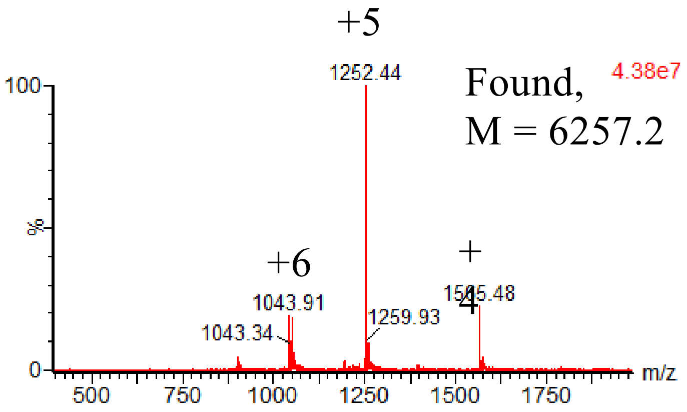

Figure S9. HPLC profile of cyclic glycopeptide **11**

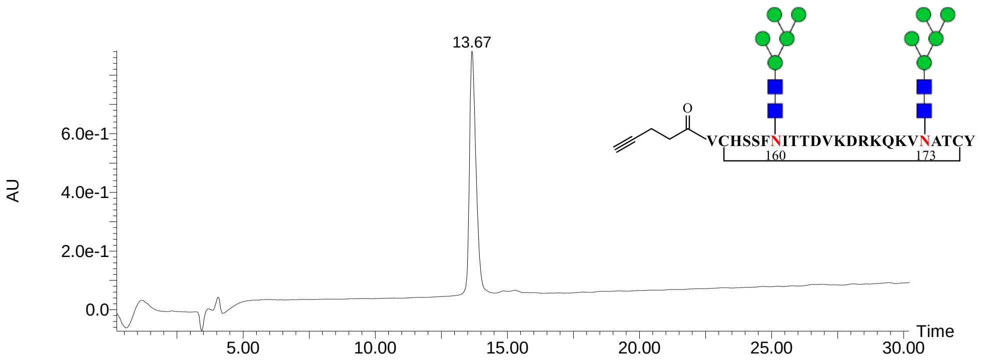

A. (UV Abs. 214 nm)

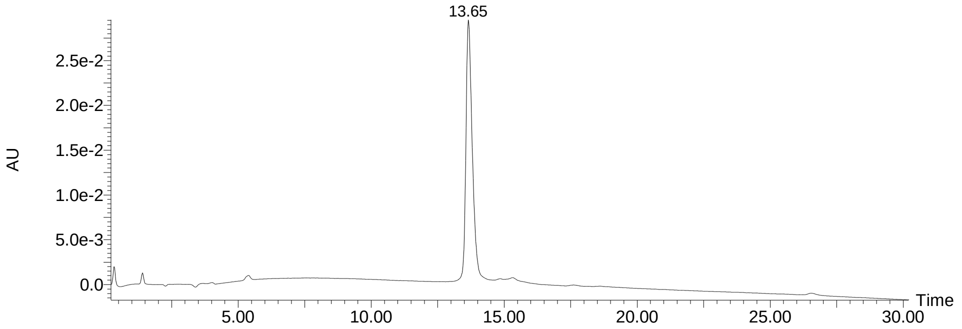

B. (UV Abs. 280 nm)

Figure S10. ESI-MS profile of cyclic glycopeptide **11**

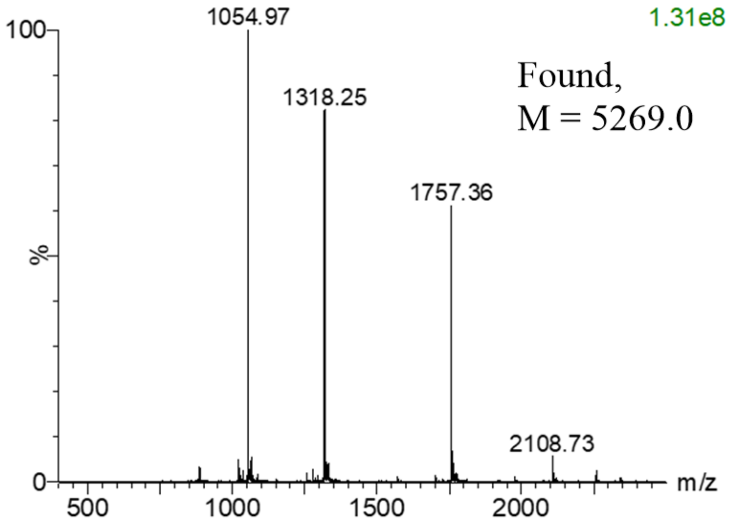

Supplement: Supplementary file 1 [file ijms-22-12538-s001.zip › ijms-1457191-supplementary.pdf]
